# Development of a predictive model to distinguish prostate cancer from benign prostatic hyperplasia by integrating serum glycoproteomics and clinical variables

**DOI:** 10.1186/s12014-023-09439-4

**Published:** 2023-11-21

**Authors:** Caterina Gabriele, Federica Aracri, Licia Elvira Prestagiacomo, Maria Antonietta Rota, Stefano Alba, Giuseppe Tradigo, Pietro Hiram Guzzi, Giovanni Cuda, Rocco Damiano, Pierangelo Veltri, Marco Gaspari

**Affiliations:** 1 Research Centre for Advanced Biochemistry and Molecular Biology, Department of Experimental and Clinical Medicine, Magna Graecia University of Catanzaro, Catanzaro, Italy; 2 Department of Surgical and Medical Sciences, Magna Graecia University of Catanzaro, Catanzaro, Italy; 3 Romolo Hospital, Rocca Di Neto, Italy; 4 Ecampus University, Novedrate, Italy; 5 Department of Experimental and Clinical Medicine, Magna Graecia University of Catanzaro, Catanzaro, Italy; 6 Department of Computer Engineering, Modeling, Electronics and Systems, University of Calabria, 87036 Rende, Italy

**Keywords:** Biomarker panel, Mass spectrometry, Machine learning, Ribonuclease pancreatic, Lysosome-associated membrane glycoprotein 2, Lumican, Mannan-binding lectin serine protease 1, Neural cell adhesion molecule 1, Phosphatidylinositol-glycan-specific phospholipase D

## Abstract

**Background:**

Prostate Cancer (PCa) represents the second leading cause of cancer-related death in men. Prostate-specific antigen (PSA) serum testing, currently used for PCa screening, lacks the necessary sensitivity and specificity. New non-invasive diagnostic tools able to discriminate tumoral from benign conditions and aggressive (AG-PCa) from indolent forms of PCa (NAG-PCa) are required to avoid unnecessary biopsies.

**Methods:**

In this work, 32 formerly N-glycosylated peptides were quantified by PRM (parallel reaction monitoring) in 163 serum samples (79 from PCa patients and 84 from individuals affected by benign prostatic hyperplasia (BPH)) in two technical replicates. These potential biomarker candidates were prioritized through a multi-stage biomarker discovery pipeline articulated in: discovery, LC-PRM assay development and verification phases. Because of the well-established involvement of glycoproteins in cancer development and progression, the proteomic analysis was focused on glycoproteins enriched by TiO_2_ (titanium dioxide) strategy.

**Results:**

Machine learning algorithms have been applied to the combined matrix comprising proteomic and clinical variables, resulting in a predictive model based on six proteomic variables (RNASE1, LAMP2, LUM, MASP1, NCAM1, GPLD1) and five clinical variables (prostate dimension, proPSA, free-PSA, total-PSA, free/total-PSA) able to distinguish PCa from BPH with an area under the Receiver Operating Characteristic (ROC) curve of 0.93. This model outperformed PSA alone which, on the same sample set, was able to discriminate PCa from BPH with an AUC of 0.79.

To improve the clinical managing of PCa patients, an explorative small-scale analysis (79 samples) aimed at distinguishing AG-PCa from NAG-PCa was conducted. A predictor of PCa aggressiveness based on the combination of 7 proteomic variables (FCN3, LGALS3BP, AZU1, C6, LAMB1, CHL1, POSTN) and proPSA was developed (AUC of 0.69).

**Conclusions:**

To address the impelling need of more sensitive and specific serum diagnostic tests, a predictive model combining proteomic and clinical variables was developed. A preliminary evaluation to build a new tool able to discriminate aggressive presentations of PCa from tumors with benign behavior was exploited. This predictor displayed moderate performances, but no conclusions can be drawn due to the limited number of the sample cohort.

Data are available via ProteomeXchange with identifier PXD035935.

**Supplementary Information:**

The online version contains supplementary material available at 10.1186/s12014-023-09439-4.

## Background

Prostate cancer (PCa) is the most frequently diagnosed neoplasia, covering about a quarter of new cancer diagnoses, and the second leading cause of cancer-related death in males [1]. The considerable mortality rate of this tumor underlines the impelling need to improve the diagnostic and therapeutic tools currently used in clinics.

Prostate-specific antigen (PSA) blood testing is often employed to select patients eligible for prostate biopsy but its routinary blood measurement in clinical check-ups is controversial by virtue of its limited specificity and sensitivity [2]. Furthermore, of no less importance is the risk of overdiagnosis and overtreatment associated with the use of this biomarker [3].

Over the years, improvements in proteomic technologies have fostered interest in mass spectrometry (MS)-based discovery of new cancer biomarkers [4]. In particular, MS is a powerful method that enables to unveil proteomes in depth and to shed light on proteomic perturbations that can play a significant role in cancer [5]. Blood proteomics has always attracted a special interest in the field of biomarker discovery. In fact, blood samples are easily collectible and widely available.

Since tissue-derived proteins are diluted in the systemic circulation, the concentration of proteins of potential interest in cancer biomarker discovery lies below a few ng/mL [6]. Besides, direct protein quantification by MS in enzymatically digested blood is further hampered by the high complexity of this biological sample, in which protein constituents differ in their concentration by several orders of magnitude [7]. This technical challenge fostered the development of enrichment strategies aimed at reducing sample complexity and at enriching the blood proteome with low abundance proteins [8]. In this regard, particularly intriguing is the strategy of glycoprotein enrichment. Glycoproteins are intended for secretion, thus they will likely be found in the systemic circulation. Moreover, most cancer biomarkers currently in use are glycoproteins [9–11]. Finally, the involvement of glycoproteins in cancer development and progression is well established [12].

Glycoproteomics of PCa is an ever-expanding field, as demonstrated by the numerous studies which belong to this area of interest [13]. An early pivotal study about PCa biomarker discovery identified a serum glycoprotein signature comprising ASPN, CTSD, HYOU1 and OLFM4, (from this point on, throughout the text, proteins will be indicated via their gene names to allow a more fluent reading) able to discriminate between BPH and PCa groups with an area under the ROC curve (AUC) of 0.726 [14]. Glycoproteomics has also been explored for discriminating between indolent PCa (NAG) and aggressive PCa (AG) through a multiplexed targeted MS assay based on parallel reaction monitoring (PRM) [15]. The implementation of leaner sample preparation workflows and the increased robustness of LC–MS methods have allowed higher throughput studies. As a result, a recent work by by Sajic et al. [16]. reported biofluid glycoproteomics of five different types of localized cancers in a large sample cohort. This multi-cancer comparison identified both tumor-specific biomarkers and “common biomarkers” reflecting the systemic response to cancer.

**Fig. 1:**
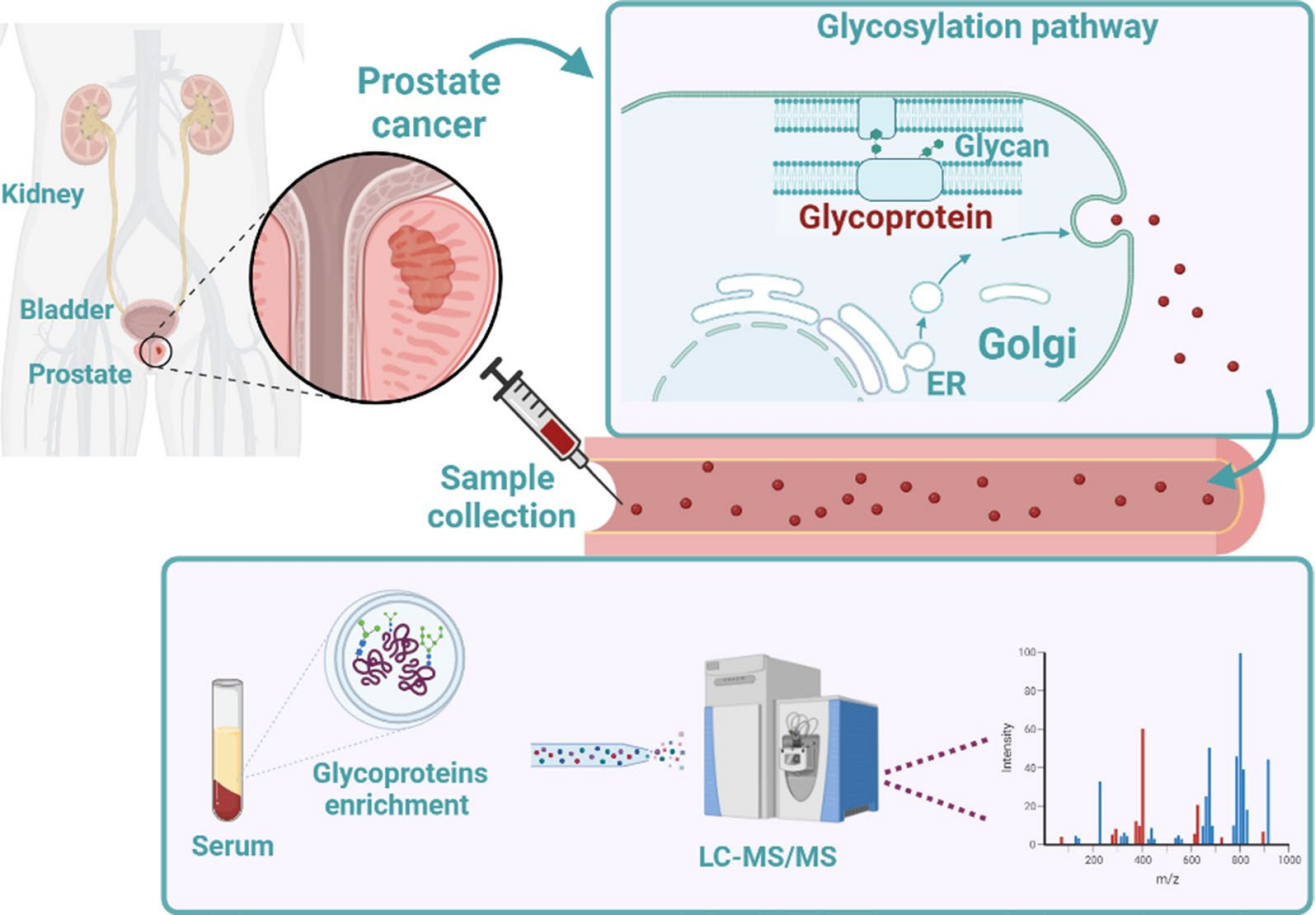
Serum glycoproteomics for PCa biomarker discovery

Here, we present the development of a predictive model able to distinguish PCa from BPH patients based on a few clinical variables combined with a panel of proteins measured by targeted-MS. The protein panel was chosen by implementing a multi-stage strategy for serum glycoproteomics articulated in a discovery, a PRM assay development and a verification phase followed by multivariate analysis (Fig. 1).

The complexity issues associated with the use of serum as biological sample were tackled by coupling extensive fractionation strategies and glycopeptide enrichment. For this purpose, a TiO_2_ (titanium dioxide) enrichment protocol as reported by Palmisano et al. was preferred because of its intrinsic suitability for automation [17]. This method allows the predominant enrichment of sialylated glycopeptides. In the verification phase, 32 selected peptides were quantified on 79 PCa and 84 BPH specimens in duplicate in MS targeted mode using isotopically labelled peptides. Then, proteomic quantitative information about these 32 peptides together with some routinely measured clinical variables were subjected to feature selection using machine learning algorithms. The application of this complex pipeline allowed the development of a predictive model which could discriminate PCa from BPH patients with an AUC (area under the curve) of 0.93. With the aim of addressing the impelling need of more informative biomarker panels of tumor aggressiveness, a preliminary attempt was made to develop such a tool.

## Methods

### Materials

All chemicals used in the experiments described in this section were purchased from Sigma-Aldrich unless otherwise specified.

### Sample collection

Blood samples were obtained from the Urology Units of Romolo Hospital (Kr) and Magna Graecia University of Catanzaro. Specimens were collected from PCa before radical prostatectomy and any therapeutic treatment, while patients suffering from BPH were recruited as controls. Inclusion criteria were: prostate biopsy performed at least 4 weeks prior to recruitment (with a minimum of 12 sampling). The exclusion criteria were: previous prostatic surgery, radiotherapy of the pelvis, neoadjuvant anti-androgenic therapy, therapy with 5-alpha reductase inhibitors.

Quantification of proPSA on serum samples was performed by enzyme-linked immunosorbent assay (ELISA) using the MYBioSource kit.

### Discovery experiments

Three different discovery experiments were carried out in order to expand as much as possible the list of potential candidates to be validated by PRM. In particular, these multiple discovery experiments were performed starting from the same 40 digested serum samples (20 PCa and 20 BPH) and are referred in the following sections as “Discovery TMT-A”, “Discovery TMT-B” and “Discovery 3D”. Discovery TMT-A and TMT-B are both based on isobaric labelling but differ in where TMT derivatization was performed in the workflow: in TMT-A, labelling was performed after glycopeptide enrichment by TiO_2_, whereas in TMT-B it preceded the enrichment procedure. On the contrary, Discovery 3D experiments were performed in label free mode to construct a database of MS spectra for verification experiments.

#### Protein digestion for discovery experiments

In-solution digestion was performed on 40 serum samples (20 PCa and 20 BPH). Briefly, 25 µL of each sample were diluted with 225 µL of 100 mM triethylammonium bicarbonate (TEAB)/2.5% sodium deoxycholate (DOC) (w/v). Then, protein disulphide bonds between cysteine residues were reduced by adding 25 µL of 100 mM dithiothreitol (DDT) and incubating the samples for 5 min at 95 °C, then for 60 min at 37 °C with gentle agitation. Cysteine residues were alkylated by 24 mM (final concentration) iodoacetamide (IAA) for 60 min at 37 °C with gentle agitation. Then, IAA excess was quenched by an extra 2 mM DTT (incubation of 30 min at 37 °C). Finally, 124 µL of each sample, corresponding to 10 µL of undiluted serum, were withdrawn and mixed with 365 µL of 50 mM TEAB in order to lower DOC concentration down to 0.5%. Samples were digested overnight with 8 µg of trypsin using a 1:100 E/S ratio (37 °C at 650 rpm).

#### ﻿Discovery TMT-A

Hundred microliters of each digested specimen (about 160 µg) were pooled in groups of 4 for a total of 10 sample pools (5 PCa and 5 BPH). DOC was removed by precipitation (Additional file 1). Then,

glycosylated peptides were enriched by the use of TiO_2_ beads following the protocol of Palmisano and co-workers [17].

The 10 sample pools were labelled by Tandem Mass Tags (TMT-10 plex, Thermo Fisher). TMT labelling was performed following the manufacturer’s protocol except for the resuspension volume of TMT reagents, which was 100 µL of anhydrous ACN (final TMT concentration of 0.8 µg/µL) (Additional file 1).

TMT-labelled sample pools were combined in 1:1 ratio into a single sample. This sample was fivefold diluted in Wash B (80% ACN/0.5% formic acid (FA) (v/v)) and then fractionated by strong cation exchange (SCX) StageTip (Additional file 1) [18]. Then, the 10% of each fraction was analyzed by nanoliquid chromatography-tandem mass spectrometry (nLC-MS/MS).

#### Discovery TMT-B

Twenty-five µL of digested samples (about 40 µg) were pooled in groups of 4 samples for a total of 10 pooled samples (5 PCa and 5 BPH). Subsequently, peptides were labelled as described in Additional file 1. After having verified that the labelling reaction was complete, by injecting a small aliquot of each sample in nLC-MS/MS prior to quenching [19], the labelling reaction was quenched by hydroxylamine. Then, all samples were combined in 1:1 ratio into a single sample mix (about 1.6 mg in a volume of 12 mL).

Labelled peptides were separated from the detergent by acid precipitation followed by solid-phase extraction (SPE) (Additional file 1).

By virtue of a higher quantity of peptide starting material, TiO_2_ enrichment was performed using 10 mg of beads. Washings and elution were performed as described in Additional file 1, section “Glycopeptide enrichment”. Then, glycopeptides were de-glycosylated by the addition of 6 µL of PNGase F (overnight incubation at 37 °C with gentle agitation).

Formerly glycosylated peptides were separated in 10 fractions by C_18_ (Empore™-3M, C_18_) StageTips performed at basic pH (Additional file 1). Then, 25% of each fraction was analyzed by nLC-MS/MS.

#### Discovery 3D

Discovery 3D experiments were performed to create a database of MS/MS spectra for the subsequent verification phase. The experiments were performed using solely PCa specimens (20 samples), since protein identification was mainly directed towards hits potentially increased in PCa. From each sample digest, obtained as previously described, 175 µL of solution were withdrawn and pooled (total volume was 3.5 mL, total peptide amount 5 mg). DOC was precipitated using TFA 0.5% and the supernatant was withdrawn and purified by SPE HLB (3 cc) as described in Additional file 1 section “High pH C_18_ fractionation”. After SPE purification, the obtained eluate was lyophilized. The SPE eluate was resuspended in Titanium Loading Buffer and glycopeptides were enriched as described in Additional file 1, section “Glycopeptide enrichment”. In this case, 25 mg of TiO_2_ beads were used. Finally, enriched glycopeptides were incubated with 10 µL of PNGase F to remove carbohydrate moieties.

Formerly N-glycosylated peptides were fractionated in 10 fractions by Basic pH fractionation as described in in Additional file 1 section “High pH C_18_ fractionation”, using an increased amount of stationary phase in order to accommodate the higher amount of material. In particular, three different StageTips, each packed with 3 Empore C_18_ disks were used. Each of the 10 fractions was divided further into 5 additional fractions by SCX using the procedure described in Additional file 1 section “Strong cation exchange (SCX) StageTip”. Fractions 7, 8 and 9 eluted from the basic pH C_18_ StageTips were combined in a single fraction because of their low peptide content. After this procedure, 40 fractions in total were obtained; 25% of each fraction was processed by nLC-MS/MS.

#### nLC-MS/MS analysis of discovery experiments

All the fractions from Discovery Experiments were analyzed by tandem mass spectrometry in data-dependent acquisition mode (DDA). Briefly, chromatographic separation was performed by nanoflow chromatography using EASY-LC-1000 instrument (Thermo Fisher) coupled with a Q-Exactive mass spectrometer (Thermo Fisher). Peptides were separated by an in-house made analytical column packed to 14 cm of length with 3 μm C_18_ silica particles (Dr. Maisch). Gradient elution was obtained using a binary gradient of 140 min at a flow rate of 300 nL/min. The mobile phase A and B were (2% ACN/0.1% FA (v/v)) and (80% ACN/0.1% FA (v/v)) respectively. The percentage of mobile phase B was increased from 0 to 6% in 1 s, then to 38% in 120 min and to 100% in 15 min. After 5 min at 100%, mobile phase B was then decreased to 0% in 2 min. MS detection of peptides gradually eluted from the analytical column, was performed by nanoelectrospray (nESI) applying a potential of 1700 V to the column front-end via a tee piece. DDA was performed by using a top-12 method, where the 12 most abundant ions were automatically selected for HCD fragmentation (collision energy was set at 34% for TMT experiments and at 25% for the Discovery 3D experiment).

Resolution, AGC target and maximum injection time (ms) for full MS and MS/MS were 70 000/35000, (1 × 10^6^)/(2 × 10^5^), 50/120, respectively. MS full scan range was 350 − 1800 m/z. Mass window for precursor ion isolation was 1.6 m*/z*. Ion threshold for triggering MS/MS events was 1 × 10^5^. Dynamic exclusion was 30 s.

#### Data analysis of discovery experiments

The raw files from TMT-A experiments were analyzed with Proteome Discoverer (v. 2.1) using Sequest HT as search engine. Search parameters were the following: MS tolerance 15 ppm; MS/MS tolerance 0.02 Da. Trypsin was selected as an enzyme and two missed cleavage sites were allowed. TMT labelling of lysines and N-terminus (+ 229.163 Da), deamidation of asparagines (+ 0.984 Da), and oxidation of methionines (+ 15.995 Da) were set as variable modifications, while carbamidomethylation of cysteines (+ 57.021 Da) was set as fixed modification. Only peptides harboring glycosylation consensus sequence (NXT/S) and fully labelled were kept for subsequent statistical analysis.

Data from TMT-B and Discovery 3D experiments were analyzed with MaxQuant (v. 6.2). The following parameters were used: enzyme trypsin, maximum 2 missed cleavages, MS tolerance 3.5 ppm after recalibration and MS/MS tolerance of 20 ppm. The dynamic modifications were: methionine oxidation (+ 15.995 Da), asparagine deamidation (+ 0.984 Da), TMT labelling of lysines and N-terminus (+ 229.163 Da) (for TMT-B). Carbamidomethylation of cysteines (+ 57.021 Da) was set as static modification.

The Human Uniprot protein sequence database accessed on 15 November 2017 was used as sequence database (20184 entries).

Statistical analysis of Discovery TMT-B data was performed with Perseus software. Protein intensities were log2 transformed and normalized based on the median value of all intensities. Differentially expressed proteins were filtered based on *p-value* < 0.1 and a fold-change > 1.1 and presence of glycosylation consensus (NX/T).

Results Discovery 3D were analyzed only from a qualitative point of view interpolating the list of identifications with BioGPS (www.biogps.org) and with candidate lists selected from the literature^16,18^ in order to identify proteins involved in PCa development.

### LC-PRM assay development

In this phase, the analytes selected in the discovery experiments were quantified in targeted mode by PRM in label free mode. Proteomic analysis was carried out on 53 specimens (27 BPH, 26 PCa). Serum samples were digested, DOC was precipitated and, then glycopeptides were enriched by TiO_2_ enrichment and purified by C_18_ as described in the section relative to the discovery experiments.

#### PRM Quantification of formerly N-linked glycopeptides

Discovery experiments resulted in a list of 34 formerly *N*-linked glycopeptides (belonging to 31 proteins) of interest for PRM quantification in label free mode in individual samples. The selected candidates and the relative proteins are illustrated in the Table 1.

**Table 1:** Candidates tested by PRM and their blood concentration according to the Human Protein Atlas.

Protein	Gene	Peptide	Charge	Experiment	Blood concentration (ng/mL)
Ribonuclease pancreatic	RNASE1	S**N**SSMHITDCR	3	discovery-3D	1100
C-type mannose receptor 2	MRC2	VTPAc**N**TSLPAQR	2	discovery-3D	36
Neutrophil elastase	ELANE	VVLGAH**N**LSR	3	discovery-3D	0.37
Pantetheinase	VNN1	MTGSGIYAP**N**SSR	2	discovery-3D	980
Neural cell adhesion molecule L1-like protein	CHL1	ISGV**N**LTQK	2	discovery-3D	5800
Neural cell adhesion molecule L1-like protein	CHL1	IIPS**N**NSGTFR	2	discovery-3D	5800
Pantetheinase	VNN1	LTGVAG**N**YTVCQK	2	discovery-3D	980
Interleukin-6 receptor subunit beta	IL6ST	LTV**N**LTNDR	2	discovery-3D	160
Lysosome-associated membrane glycoprotein 2	LAMP2	VQPF**N**VTQGK	2	discovery-3D	520
Ficolin-3	FCN3	VELEDFNG**N**R	2	discovery-3D	18000
Cathepsin D	CTSD	GSLSYL**N**VTR	2	discovery-3D	370
Metalloproteinase inhibitor 1	TIMP1	FVGTPEV**N**QTTLYQR	2	discovery-3D	110
Azurocidin	AZU1	FV**N**VTVTPEDQCRPNNVCTGVLTR	3	discovery-3D	0.32
Lactotransferrin	LTF	**N**GSDCPDKFCLFQSETK	3	discovery-3D	350
Adipocyte plasma membrane-associated protein	APMAP	AGP**N**GTLFVADAYK	2	discovery-3D	130
Periostin	POSTN	EV**N**DTLLVNELK	2	discovery-3D	660
Chondroitin sulfate proteoglycan 4	CSPG4	LDPTVLDAGELA**N**R	2	discovery-3D	48
Mannan-binding lectin serine protease 1	MASP1	N**N**LTTYK	2	discovery-3D	9400
Afamin	AFM	YAEDKF**N**ETTEK	2	TMT-A	47000
Beta-2-glycoprotein 1	APOH	VYKPSAG**N**NSLYR	3	TMT-A	280000
Plasma kallikrein	KLKB1	GVNF**N**VSK	2	TMT-A	29000
Galectin-3-binding protein	LGALS3BP	GL**N**LTEDTYKPR	3	TMT-A	7100
Lumican	LUM	LHINHN**N**LTESVGPLPK	2	TMT-A	29000
Serum paraoxonase/arylesterase 1	PON1	HA**N**WTLTPLK	2	TMT-A and B	79000
Complement component C6	C6	VL**N**FTTK	2	TMT-A	45000
Laminin subunit beta-1	LAMB1	LSDTTSQS**N**STAK	2	TMT-B	250
Receptor-type tyrosine-protein phosphatase eta	PTPRJ	S**N**DTAASEYK	2	TMT-B	670
Pregnancy-zone protein	PZP	QEVCEEFSQQLNS**N**GCITQQVHTK	4	TMT-B	15000
Pregnancy-zone protein	PZP	TFSSMTCASGA**N**VSEQLSLK	3	TMT-B	15000
Endoglin	ENG	Q**N**GTWPR	2	TMT-B	280
Neural cell adhesion molecule 1	NCAM1	**N**ISSEEK	2	TMT-B	2300
Phosphatidylinositol-glycan-specific phospholipase D	GPLD1	NI**N**YTER	2	TMT-B	110000
Uromodulin	UMOD	QDF**N**ITDISLLEHR	3	TMT-B	66
Transferrin receptor protein 1	TFRC	DFEDLYTPV**N**GSIVIVR	2	TMT-B	1300

Glycosylation site is in bold + underlined (N)

#### LC–MS method settings (PRM) and data analysis

Formerly N -linked glycopeptides were analyzed using the method described in Additional file 1 section “LC-PRM acquisition method”.

Data sets were imported into Skyline v. 19.1 and peaks were automatically integrated and manually inspected. For the quantification of the 34 selected peptides (35 precursors, since one peptide was also detected in its oxidized form), MS/MS spectra from 3D Discovery Experiments were used to build a spectral library of TiO_2_-enriched serum. The charge states of precursors were set to 2, 3 and 4, and the product ions were 1-, 2- and 3-charged (ion types y, b, p) with a up to 6 product ions. The ion match tolerance was set to 0.05 m*/z* [20].

#### Preparation of heavy peptides for validation experiment

The Heavy peptides containing either ^13^C_6_ + ^15^N_2_ atoms (Lys) or ^13^C_6_ + ^15^N_4_ atoms (Arg) at the carboxy terminal amino acid were bought from JPT Peptide Technologies (Berlin, Germany, Additional file 2: Table S1). These peptides were solubilized in 40% ACN /0.1% FA v/v; for most hydrophobic peptides, 70% ACN instead of 40% ACN was used (Additional file 2: Table S1). To test their purity and to optimize chromatographic conditions, heavy peptides were individually injected in nLC-MS/MS. After the completion of PRM verification experiments, a “heavy” peptide mixture (HPM) matching the expected relative concentrations of endogenous peptides was created. In order to obtain the HPM, concentrated peptide solutions were diluted in 40% ACN/0.1% FA until a concentration value 100-fold higher than what reported in Table 2 Si was obtained; resulting peptide solutions were then mixed together and diluted 100-fold in 10% ACN/0.1% FA. HPM was stored in 10 µL aliquots at -80 °C until its use as internal standard. The concentration of each heavy peptide in the HPM is illustrated in Table S2 (Additional file 2). HPM was added to each sample after protein digestion and N-glycopeptide enrichment, thus before C_18_ purification and nLC-MS/MS.

**Table 2:** Principal 11 significant variables after feature selection

Feature	Pearson	Chi-2	RFE	Logistics	Random forest	Total
ftPSA	True	True	True	True	True	5
Prostate dimension (cc)	True	True	True	True	True	5
ProPSA	True	True	True	True	True	5
tPSA	True	True	True	False	True	4
fPSA	True	True	True	False	True	4
VQPFNVTQGK	True	True	True	True	False	4
SNSSMHITDCR	True	True	True	False	True	4
NNLTTYK	True	True	True	True	False	4
NINYTER	True	True	True	True	False	4
LHINHNNLTESVGPLPK	True	True	True	True	False	4
DGQLLPSSNYSNIK	True	True	True	True	False	4

### Verification experiments

This phase was focused on the analysis by PRM of the selected candidates by nLC-MS/MS in targeted mode by using isotopically labelled peptides as internal standards. This subset of experiments was performed in duplicates on an independent subset of 79 PCa and 84 BPH patients.

#### Ultimate sample processing workflow and PRM analysis

Samples were processed as described in the discovery experiments, making only minor changes to the original protocol (Additional file 1 section “Sample processing workflow”).

The whole pipeline was carried out in duplicate for 84 BPH and 79 PCa serum samples, accomplishing a total of 326 nLC-MS/MS analyses.

nLC-MS/MS analysis was performed using the acquisition method described in Additional file 1 section “Ultimate LC-PRM acquisition method”.

A schematic view of the PRM method is reported in Additional file 2: Table S3.

#### Data analysis

The variability of the glycopeptide enrichment procedure was corrected through introducing a normalization factor based on the quantification, by extracted ion chromatogram (XIC), of 30 highly abundant serum glycopeptides (Additional file 2: Table S4). The selection criteria for the 30 glycopeptides used for normalization were: high concentration and no involvement in inflammation.

Sample replicates were evaluated for their concordance. As criterion, the “scaled relative difference” (SRD) introduced by Hyslop and White in 2009 was chosen [21]. The scaled relative difference can be defined by the following formula: (C_i1_-C_i2_)/C_i_√2

where C_i1_ and C_i2_ represent the sum of all XIC values for the 30 reference glycopeptides in replicates 1 and 2, respectively, whereas C_i_ is the average of the two measures. SRD higher than 0.50 (or lower than − 0.50), indicating a difference in glycopeptide abundance between the two replicates higher than twofold, was considered not acceptable. In this case, the replicate with the lower recovery of glycopeptides was discarded. Besides, nLC-MS/MS runs having a C_i1_ or C_i2_ value 2 standard deviations lower than the average C_i_ in the data set were also excluded (Additional file 3: Table S5). After this preliminary filtering operations, 131 duplicate analyses and 32 single analyses, respectively, were subjected to multivariate analysis.

#### Multivariate analysis

The calculated areas of light peptides were corrected by the IS signal (heavy peptide) as follows:

L*n*’ = *f* * L*n.*

where L*n* is the area obtained for the n-th peptide light, L*n*’ is the corrected area, and f is the correction factor obtained with the formula below:

*f* = H*m* / H*n.*

where Hn is the area of the n-th peptide (heavy form) and Hm is the average value of the n-th heavy peptide in the overall sample set.

After being corrected, the areas of endogenous peptides were normalized using the normalization factor, considering the enrichment efficiency. Peptide areas were divided by: C_i1 (or 2)_ / C_a_, were C_i1_ and C_i2_ represent the sum of all XIC values for the 30 reference glycopeptides in replicate 1 and 2, respectively, whereas C_a_ represents the average value obtained for the entire data set. Finally, for each sample having a technical duplicate, the average between the two replicates for each peptide was calculated. The whole data matrix after peptide normalization is reported in Table S6 (Additional file 4).

Clinical variables (Additional file 4: Table S7) together with mass spectrometric results (32 peptide areas) were filtered by feature selection exploiting different approaches. In particular, Random Forest, Chi-square test, Pearson coefficient, Lasso regression and Recursive feature elimination have been used. According to each model’s metrics, the feature selection identifies the most statistically important characteristics and ranks them according to relevance score. The linear correlation between two attributes is measured by the Pearson correlation coefficient [22]. The Pearson correlation coefficient given two random variables X and Y, is the ratio of their covariance to the sum of their respective standard deviations. To test the independence of two events, the Chi-square is used [23]. The test examines the difference between the observed count and predicted count given two factors. The observed count is close to the expected count when two variables are independent, which lowers the Chi-square value. The Recursive Feature Elimination (RFE) [24] is then added to the feature selection module in order to fit the model and eliminate the worst features. By iteratively removing features, the RFE enables to decrease the collinearity that already exists in the supplied data. RFE enables us to recursively reduce features by examining data that show their relative relevance. Random Forest (RF) ensures good data abstraction results also because it is easy to calculate the relative value of each feature on the produced decision tree. Several random decision trees with nodes containing binary questions depending on a single or a combination of features are generated by RF. The tree splits the dataset into two subsets at each node. The effectiveness of each feature, or group of features, in dividing the dataset is then taken into account when determining its relevance. For each test, the maximum number of significant variables has been limited to 20. Table 2 illustrates, in decreasing order of significance, the 11 variables of higher interest.

The list was filtered leaving variables significant for at least 4 of the algorithms (i.e. the first 11 variables) giving the following set: pro-PSA, Free PSA/Total PSA, Gland volume, Total PSA, Free PSA, VQPFNVTQGK (LAMP2), NINYTER (GPLD1), LHINHNNLTESVGPLPK (LUM), DGQLLPSSNYSNIK (NCAM1), SNSSMHITDCR (RNASE1), NNLTTYK (MASP1). Then, the sample set was divided into two groups: 143 samples were used to build the predictive model, whereas the remaining 20 samples were used to evaluate the performance of the model by using a voting strategy [25].

In particular, concerning model creation, 100 out of 143 samples were used as training set (70% of the dataset) and the remaining 43 samples (30% of the dataset) as testing set. The algorithm showing the highest predictive performance (Random Forest), was selected by considering the highest AUC and also the highest sensitivity score, which is one of the most relevant measures in clinical applications, since it gives an idea of the ability of the model to minimize false negatives.

Lastly, to evaluate the performance of the predictive model, ML algorithms results were integrated by implementing a voting strategy. In particular, two voting strategies were employed to merge the outcomes of all the algorithms: hard voting and soft voting. Hard voting counted ML models that agreed on the predicted classes. More specifically, if 4/5 ML models agreed on PCa for a certain input, the hard voting strategy returned PCa as result. Whereas, concerning the soft voting approach, each ML model prediction (i.e., PCA or BPH class) was weighted by the F1 performance measure. The voting strategy was applied for the classification of 20 patients belonging to the diagnostic grey zone (tPSA 4–10 ng/mL).

A separate data analysis focused only on PCa dataset was also conducted to evaluate the possibility to distinguish high grade (AG-PCa) from low grade tumors (NAG-PCa). PCa sample set was divided in two subgroups: 53 AG-PCa (Gleason > 3 + 3) and 26 NAG-PCa (Gleason 3 + 3). The principal contributing variables were triaged by feature selection step. Model testing was performed on 55 samples (70% of the PCa sample set). The selected variables, ranked on their relative contribution to the model, were: FCN3, proPSA, LGALS3BP, AZU1, C6, LAMB1, CHL1, POSTN. The model was tested with the remaining 30% of the sample set (24 data samples).

## Results

**Fig. 2:**
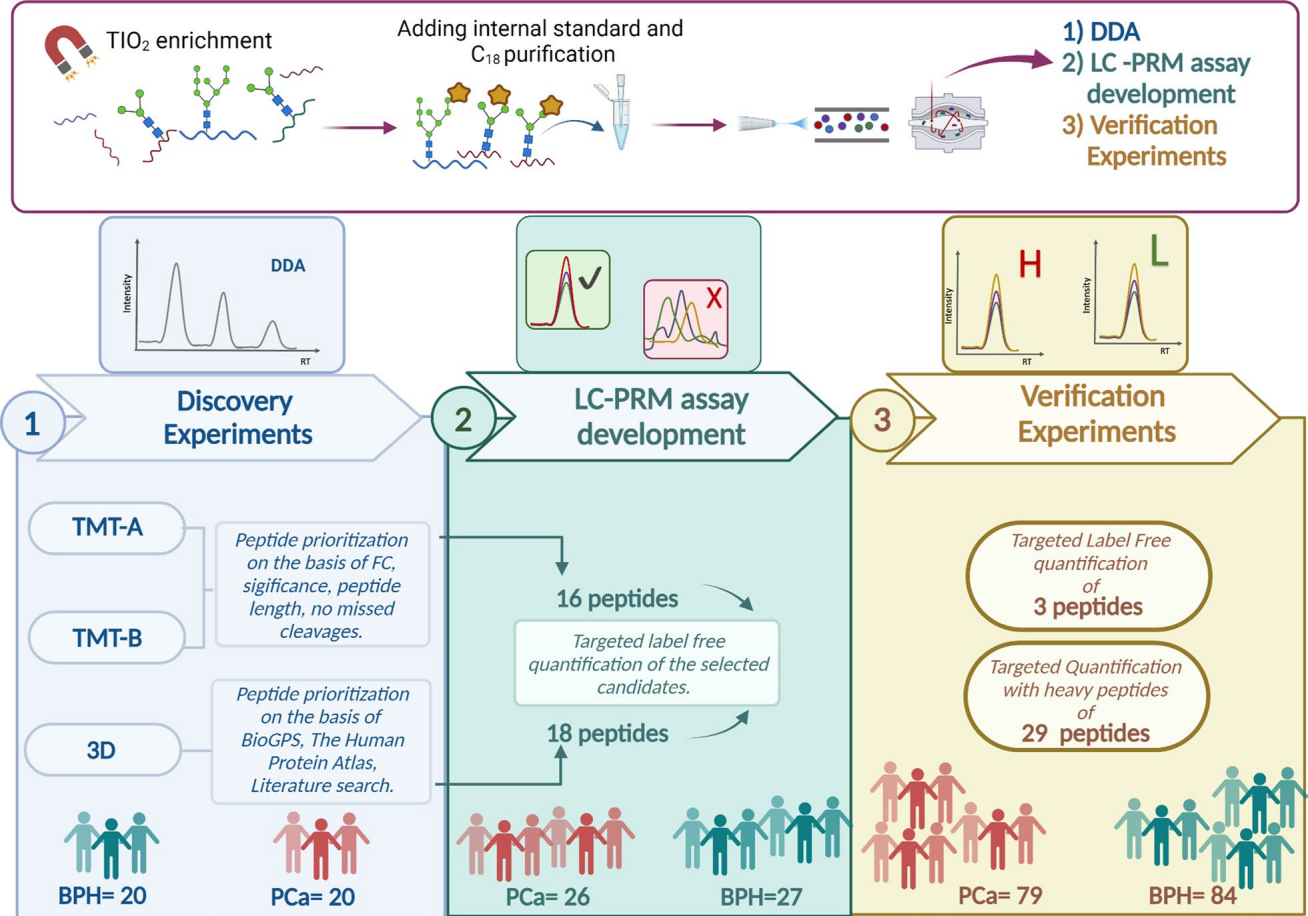
Proteomic biomarker discovery pipeline

The primary goal of this work was to develop a predictive model able to discriminate between PCa and BPH patients based on a combination of clinical and proteomic variables. Proteomic data were generated by a multistage biomarker discovery effort articulated in three distinct phases: discovery, LC-PRM assay development, and verification. The complete proteomic biomarker discovery pipeline is depicted in Fig. 2.

### Discovery phase

The discovery phase was carried out on a limited number of samples (20 BPH and 20 PCa) using high amounts of starting material and implementing strategies to de-complex the proteome of serum samples (creation of sample pools, fractionation protocols and glycopeptide enrichment by TiO_2_) to maximize the opportunity of retrieving tumor-derived proteins in blood.

Two separate TMT-based discovery approaches were employed. In the first one (TMT-A), sample pooling was followed by detergent precipitation, TiO_2_ enrichment, and TMT labelling. The advantage of this workflow was the limited consumption of the relatively expensive TMT reagent. In the second discovery experiment (TMT-B), TMT labelling was performed soon after protein digestion in order to further reduce technical variability. In both TMT workflows, the enriched glycopeptides were de-glycosylated by PNGase F prior to offline fractionation and LC–MS/MS analysis. Since proteins can have multiple glycosylation sites and the extent of glycosylation can vary from site to site, we decided to perform statistical analysis at the peptide level. Comparative analysis of formerly N-glycosylated peptides in the cancer pools and in relative controls were evaluated by performing a Student’s t-test (*p-value* < 0.1). In the light of the fact that our interest was focused on proteins increased in the cancer group [26], we retrieved from both experiments peptides significantly increased in PCa by at least a factor of 1.2. We applied relaxed criteria for the initial selection of candidates since the LC-PRM assay used in the subsequent phases of our biomarker discovery pipeline could provide robust quantification of tens of different analytes.

Seven significant peptides from TMT-A experiment met the aforementioned selection criteria (Table 3).

**Table 3:** List of candidates selected after TMT-A experiment with relative fold-changes (FC).

Gene	Master protein	Annotated sequence	FC	Charge	*p-value*
KLKB1	P03952	GVNF**N**VSK	2.42	2	0.026
C6	P13671	VL**N**FTTK	2.13	2	0.013
AFM	P43652	YAEDKF**N**ETTEK	2.11	2	0.006
APOH	P02749	VYKPSAG**N**NSLYR	1.81	3	0.005
LGALS3BP	Q08380	GL**N**LTEDTYKPR	1.73	3	0.055
PON1	P27169	HA**N**WTLTPLK	1.48	2	0.077
LUM	P51884	LHINHN**N**LTESVGPLPK	1.31	2	0.097

Glycosylation site is in bold + underlined (N)

On the other hand, TMT-B experiment resulted in 15 significant peptides exhibiting a fold-change > 1.2. Of these, only 6 precursors were included in the validation list. Nine peptides were excluded for various reasons: (a) peptide length > 25 amino acids (shorter peptides were preferred, being more easily detectable in our chromatographic conditions); (b) presence of missed cleavages; (c) proven involvement of the corresponding protein in inflammation/coagulation; (d) absence of glycosylation consensus (NXT/S). The higher precision of TMT-B, because of earlier sample mixing, resulted in several significant hits having fold-change values lower than 1.2. These hits were prioritized based on documented involvement in PCa development. After prioritization, 4 additional peptides with fold-change > 1.08 and < 1.2 were included in the list of precursors for targeted experiments deriving from experiment TMT-B, which comprised 10 peptides (Table 4). The peptide HANWTLTPLK (PON1) was selected from both TMT experiments, thus the two TMT-based discovery experiments provided a total of 16 candidates.

**Table 4:** Candidates selected after TMT-B experiment with relative fold-changes (FC)

Gene	Master protein	Annotated sequence	FC	Ref	Charge	*p-value*
PZP	P20742	QEVCEEFSQQLNS**N**GCITQQVHTK	1.53		4	0.052
PZP	P20742	TFSSMTCASGA**N**VSEQLSLK	1.41		3	0.077
ENG	P17813	Q**N**GTWPR	1.22		2	0.014
UMOD	P07911	QDF**N**ITDISLLEHR	1.21		3	0.085
PON1	P27169	HA**N**WTLTPLK	1.20		2	0.096
TFRC	G3V0E5	DFEDLYTPV**N**GSIVIVR	1.20		2	0.095
LAMB1	P07942	LSDTTSQS**N**STAK	1.15	[27]	2	0.090
NCAM1	H7BYX6	**N**ISSEEK	1.11	[14]	2	0.082
GPLD1	P80108	NI**N**YTER	1.11	[28]	2	0.098
PTPRJ	Q12913	S**N**DTAASEYK	1.09	[29]	2	0.031

Glycosylation site is in bold + underlined (N). For precursors having a FC < 1.2, the corresponding Reference used for their selection is cited in the “Ref.” column

Overall, 410 peptides belonging to 163 proteins were identified in experiment TMT-A, whereas 1188 peptides belonging to 343 proteins were identified in experiment TMT-B (Additional file 5). The first workflow was more cost-effective but was expected to deliver lower overall precision. Indeed, median CV of peptides detected in experiment TMT-A was 25% (PCa and BPH groups were considered separately). On the other hand, the second workflow provided a far better precision (median CV of 10%) though it required a much higher amount of TMT reagent. Indeed, many more significant differences were observed in experiment TMT-B. In view of these data, the workflow TMT-B, comprising isobaric labelling before TiO_2_ enrichment, should be the preferred approach.

To prepare for targeted experiments directed towards unlabeled peptides, a comprehensive spectral library of formerly N-glycosylated precursors was built by performing a large-scale, data-dependent experiment with extensive fractionation on a sample pool processed as follows. About 5 mg of proteins obtained from 20 PCa serum samples were digested and subjected to TiO_2_ enrichment, as described in the Experimental Section. Peptides were divided into 40 fractions by a combination of sequential offline basic-pH C_18_ and SCX fractionation; all fractions were analyzed by LC–MS/MS analysis. This effort resulted in the identification of 444 proteins, many of which were known to be present in serum at low abundance levels. Before proceeding with the LC-PRM assay development, we assessed whether some of these low abundance proteins had been previously associated with PCa progression by relying on: (i) BioGPS (www.biogps.org), (ii) previously published reports in the PCa field [16, 30], and (iii) in-house produced data from proteomic analysis of EPS-urine (expressed prostatic secretions) [25]. This investigation resulted in the expansion of the candidate list for targeted experiments by additional 18 peptides (Table 5).

**Table 5:** List of candidates selected in the 3D Experiment.

Gene	Master protein	Protein name	Annotated sequence	Ref	Charge
APMAP	Q9HDC9	Adipocyte plasma membrane-associated protein	AGP**N**GTLFVADAYK	[30]	2
AZU1	P20160	Azurocidin	FV**N**VTVTPEDQCRPNNVCTGVLTR	[25]	3
CHL1	O00533	Neural cell adhesion molecule L1-like protein	ISGV**N**LTQK	BioGPS	2
CHL1	O00533	Neural cell adhesion molecule L1-like protein	IIPS**N**NSGTFR	BioGPS	2
CSPG4	Q6UVK1	Chondroitin sulfate proteoglycan 4	LDPTVLDAGELA**N**R	BioGPS	2
CTSD	P07339	Cathepsin D	GSLSYL**N**VTR	[30]	2
ELANE	P08246	Neutrophil elastase	VVLGAH**N**LSR	[25]	3
FCN3	O75636	Ficolin-3	VELEDFNG**N**R	[16]	2
IL6ST	P40189	Interleukin-6 receptor subunit beta	LTV**N**LTNDR	[30]	2
LAMP2	P13473	Lysosome-associated membrane glycoprotein 2	VQPF**N**VTQGK	[30]	2
LTF	P02788	Lactotransferrin	**N**GSDCPDKFCLFQSETK	[25]	3
MRC2	Q9UBG0	C-type mannose receptor 2	VTPAC**N**TSLPAQR	[16]	2
POSTN	Q15063	Periostin	EV**N**DTLLVNELK	[30]	2
RNASE1	P07998	Ribonuclease pancreatic	S**N**SSMHITDCR	[16]	3
TIMP1	P01033	Metalloproteinase inhibitor 1	FVGTPEV**N**QTTLYQR	[30]	2
VNN1	O95497	Pantetheinase	MTGSGIYAP**N**SSR	BioGPS	2
VNN1	O95497	Pantetheinase	LTGVAG**N**YTVCQK	BioGPS	2
MASP1	P48740	Mannan-binding lectin serine protease 1	N**N**LTTYK	BioGPS	2

Glycosylation site is in bold + underlined (N)

### LC-PRM assay development

The discovery experiments were followed by the LC-PRM assay development phase performed on 53 samples (27 BPH and 26 PCa) in which the objectives were: (i) the optimization of chromatographic conditions and scanning parameters for each of the 34 selected candidates; (ii) the verification of the consistent detection and quantification of each candidate in a sufficient number of samples; (iii) the measurement of median peptide area for each of the 34 candidates, in order to design a reference heavy peptide mix containing the appropriate amount of each internal standard. A few peptides did not pass these assay development criteria, and were not detected during targeted experiments: the peptides QEVCEEFSQQLNSNGCITQQVHTK and TFSSMTCASGANVSEQLSLK belonging to PZP and the peptide QNGTWPR from ENG. These three were excluded from the final list of candidates. In this phase, we also tested the characteristics of heavy peptides and optimized relative sample mixing. Solubilization problems were encountered for heavy peptides FVDVTVTPEDQCRPNNVCTGVLTR (AZU1) and LHINHNDLTESVGPLPK (LUM). Some hydrophobic peptides may be effectively solubilized in a concentrated peptide mixture (such as digested serum sample, even after glycopeptide enrichment), but may precipitate when dissolved in plain solvent such as a water/organic mixture. On the other hand, peptide NISSEEK (NCAM1) could not be consistently detected because its extreme hydrophilicity compromised its peak shape, and consequently compromised sensitivity of detection. Probably the hydrophobic TMT tag had favored its detection in the discovery phase. This last peptide was replaced with another glycopeptide belonging to the same protein: DGQLLPSSNYSNIK. In total, 29 precursors were selected for the verification phase to be quantified by an internal standard and 3 peptides FVDVTVTPEDQCRPNNVCTGVLTR (AZU1), LHINHNDLTESVGPLPK (LUM) and DGQLLPSSNYSNIK (NCAM1) were selected to be quantified in targeted label-free mode.

### Verification phase

The last step of the proteomic analysis was the verification phase, in which candidates were quantified by PRM by relying, in most cases, on heavy peptides for accurate quantification. In total, 163 digests from patient sera (84 BPH and 79 PCa) were processed and analyzed in duplicates. In particular, digests of serum proteins were subjected to glycopeptide enrichment by TiO_2_, de-glycosylation by PNGase F, the addition of HPM, and finally peptide desalting using C_18_ stage-tips. Baseline characteristics of the entire cohort are stated in Table 6.

**Table 6:** Baseline characteristics of the entire cohort. Total PSA (tPSA), free/total PSA (ftPSA)

Clinical variables	PCA (n = 79)	BPH (n = 84)	*p-value*
Age (years), median (IQR)	69 (64.5–73.5)	69 (62–72.5)	0.13
Prostate dimension (cc), median (IQR)	40 (30–50)	55 (40–78)	< 0.05
ProPSA, median (IQR)	553.9 (385.7–996)	257.7 (87–440)	< 0.05
tPSA, median (IQR)	8.05 (5.66–15.85)	2.4 (0.93–4.84)	0.09
ftPSA, median (IQR)	16 (12–22)	35 (24–45)	< 0.05
fPSA, median (IQR)	1.43 (0.99–2.7)	0.81 (0.28–1.72)	0.23
Gleason 6, n (%)	26 (33%)	N/A	
Gleason 7 (3 + 4), n (%)	22 (28%)	N/A	
Gleason 7 (4 + 3), n (%)	16 (20)	N/A	
Gleason ≥ 8, n (%)	15 (19)	N/A	

**Fig. 3:**
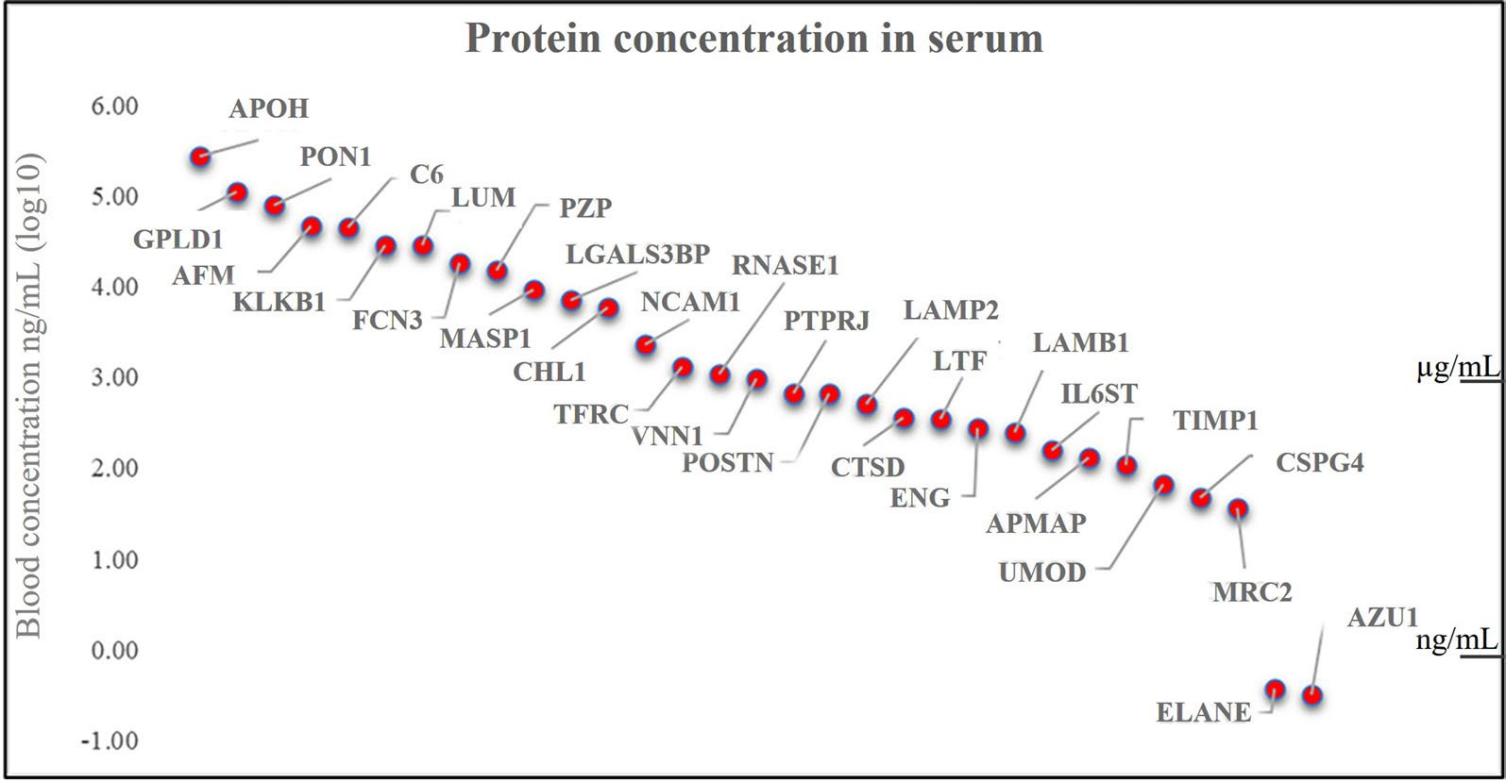
Blood concentration ng/mL (log_10_ transformed) of candidates quantified by PRM

Thirty-two peptides belonging to 30 proteins spanning a dynamic range of over 5 orders of magnitude (62 precursors in total) were quantified in a multiplexed MS analysis lasting 60 min (Fig. 3). Overall, 326 nLC-MS/MS runs were performed.

Peaks were integrated by Skyline software and manually inspected. MS runs were normalized for glycopeptide enrichment efficiency. Indeed, this step of the protocol, (TiO_2_ enrichment), being performed before the addition of “heavy” internal standards, represents the procedure harboring the major source of variability. The use of HPM before TiO_2_ glyco-capture was hampered by the impossibility of synthesizing heavy peptides bearing the full glycan structures. The variability of the enrichment step was corrected through introducing a normalization factor based on the quantification, by extracted ion chromatogram (XIC) of 30 selected high-abundance serum glycopeptides (Additional file 1: Table S4 and Additional file 2: Table S5). We assumed that the sum of the XICs of these 30 peptides was directly proportional to the overall glycopeptide recovery from TiO_2_ enrichment. Duplicate analyses were excluded when considered divergent according to the principle of “scaled relative difference” (as described in the Experimental section). Besides, single analyses having a glycopeptide content much lower than the average (lower than 2SD) were also excluded. After this preliminary selection, 131 duplicate analyses and 32 single analyses (for a total of 294 nLC-MS/MS runs) underwent multivariate statistics. Peptide peak areas were corrected by relaying on IS and on total glycopeptide content as described in the Experimental. Peak areas from duplicate analyses were averaged.

**Fig. 4:**
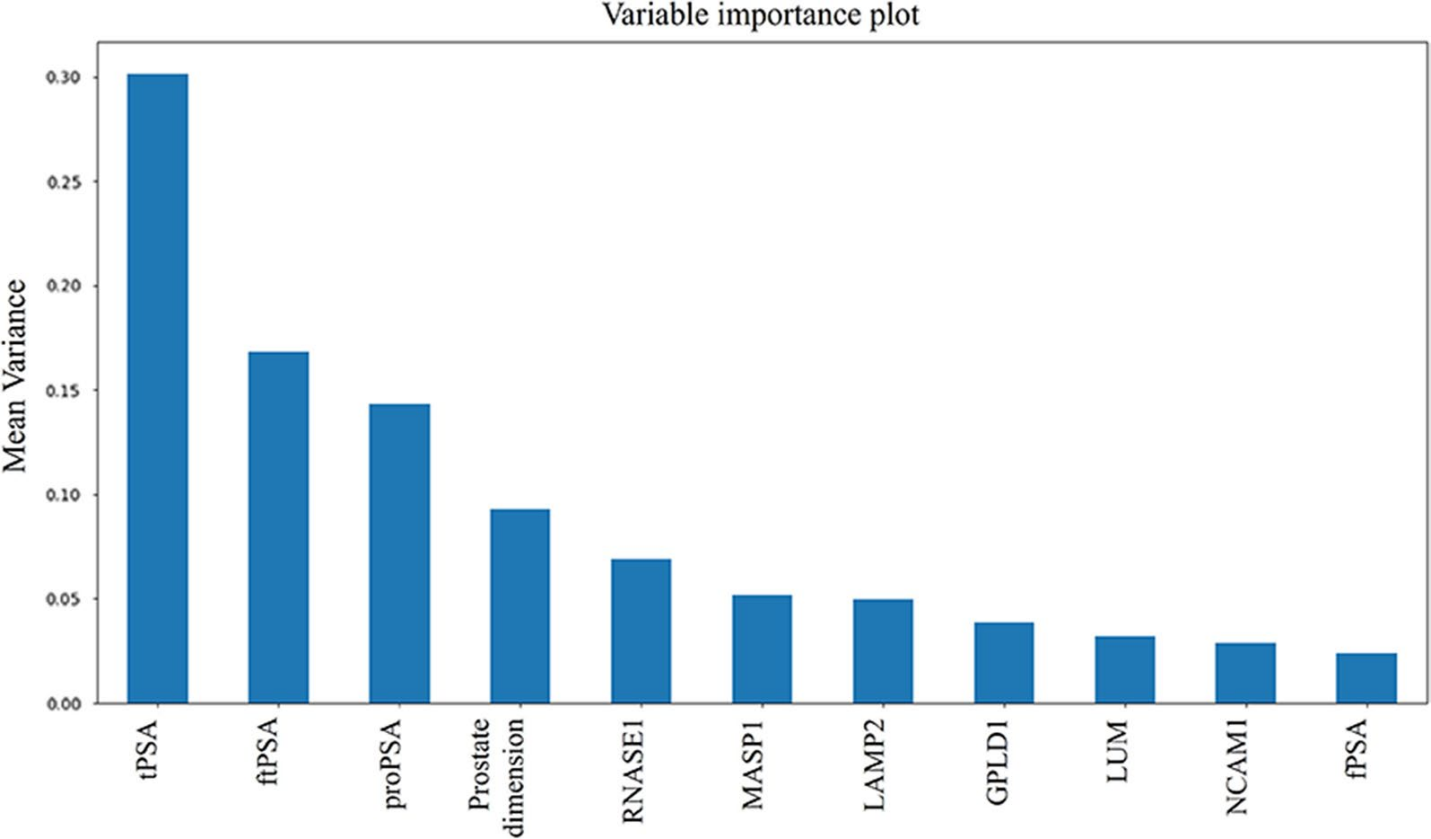
Variables constituting the predictor model plotted in decreasing order of importance (mean variance)

As reported above, the process of data analysis was conducted by considering both proteomic results and clinical information. The predictor model building was performed on approximately 90% of the sample set (143/163 samples), while about 10% of samples (20 patients having tPSA between 4 and 10 ng/mL) were intended for further evaluation of the model’s performance by the use of voting strategy [25]. Model realization, in a first step, consisted in a feature selection phase. Then, the 11 relevant ranked variables were used for model testing on 100 samples (70% of the sample set). The 11 variables, ranked in Fig. 4 based on their relative contribution to the model, were: tPSA, ftPSA ratio (free/total PSA), proPSA, prostate gland dimension, RNASE1, LAMP2, GPLD1, LUM, NCAM1, free PSA(fPSA). The model was tested with the remaining 30% of the sample set (43 data samples). For each tested algorithm, standard performance metrics have been considered (i.e. accuracy, F1, AUC, specificity and sensitivity). Results showed that the best performing model was Random Forest, which could discriminate between PCA and BPH with an AUC 0.93 (95% confidence interval, CI, 0.88–0.98) (Table 7).

**Table 7:** Standard performance metrics for the five Machine Learning models

Algorithm	AUC	f1	Accuracy	Specificity	Sensitivity
Random forest	0.93 (0.88–0.98)	0.92 (0.87–0.98)	0.93 (0.88–0.98)	0.86 (0.79–0.93)	1
Logistic regression	0.79 (0.71–0.87)	0.79 (0.71–0.87)	0.79 (0.71–0.87)	0.81 (0.73–0.89)	0.77 (0.69–0.85)
KNN	0.81 (0.74–0.89)	0.81 (0.73–0.89)	0.81 (0.74–0.89)	0.81 (0.73–0.89)	0.82 (0.74–0.89)
SVM	0.52 (0.43–0.62)	0.09 (0.03–0.15)	0.54 (0.44–0.63)	0.05 (0.01–0.09)	1
Decision tree	0.75 (0.66–0.83)	0.78 (0.70–0.86)	0.74 (0.66–0.83)	0.95 (0.91–0.99)	0.55 (0.45–0.64)

**Fig. 5:**
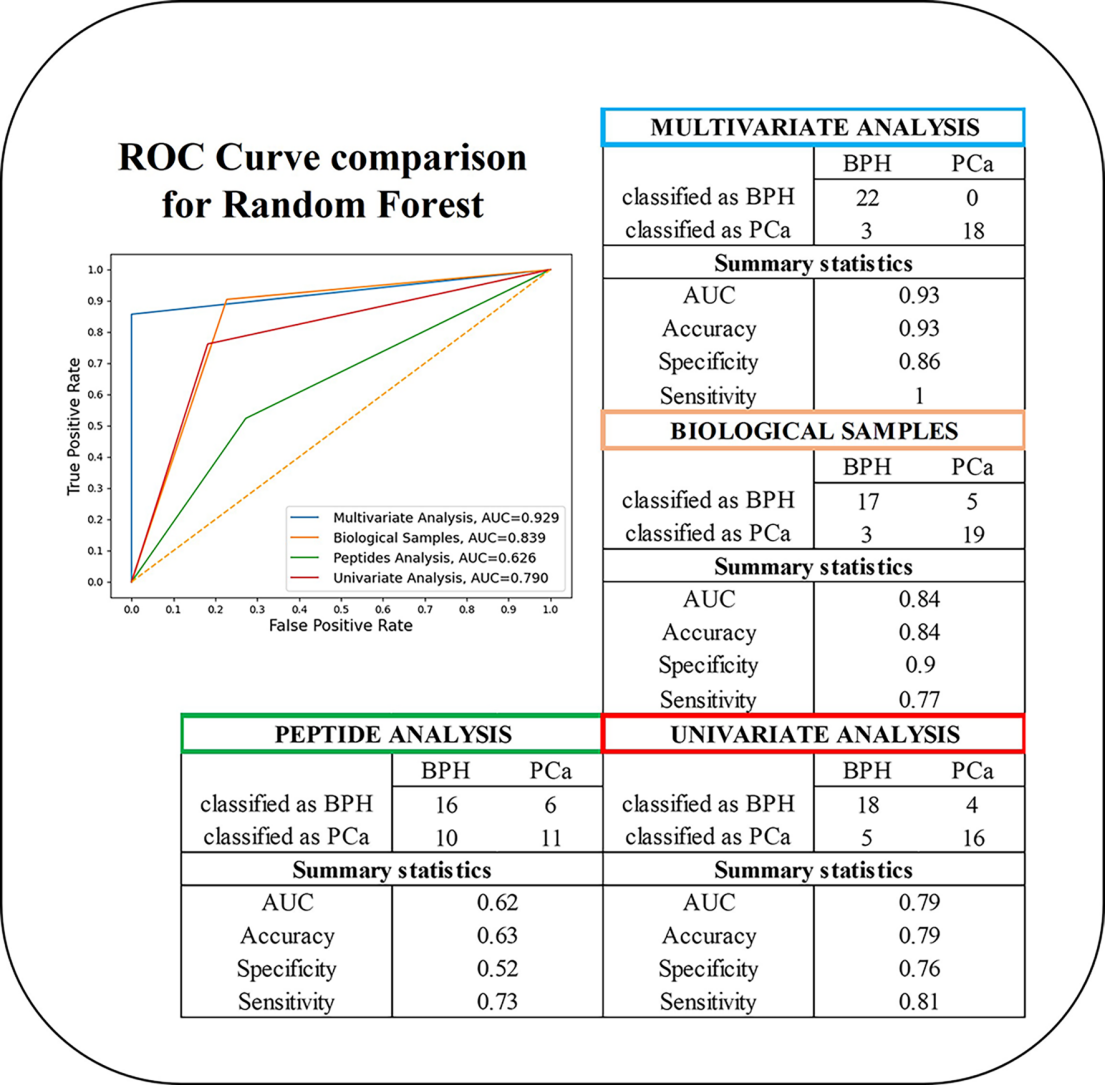
ROC curves and confusion matrix for Random Forest analysis. The plots were drawn considering the following sets of variables: (i) proteomic + clinical (named “Multivariate Analysis”, blue), (ii) proteomic variables only (named “Peptides Analysis”, green), (iii) clinical variables only (named “Biological Samples”, red), (iv) PSA only (named “Univariate Analysis”, red)

Figure 5 displays ROC curve comparison for Random Forest. The corresponding plots relative to the other four models are reported in Additional file 6. As it can be seen in Fig. 5, the multivariate model displayed AUC values higher than the univariate approach (based on tPSA). According to De-Longs test, the difference in AUC between Multivariate model and Univariate model had a p-value of 0.055. Lastly, model performance was furtherly assessed by implementing a voting strategy on 20 samples. This approach allows to merge the results of all the ML algorithms for patient’s classification and resulted in the correct assignment of 17/20 patients.

### Exploring the possibility of distinguishing between AG-PCa and NAG-PCa

PCa with Gleason score 3 + 3 (NAG-PCa) is considered a low-risk disease. By virtue of its indolent behavior, this subgroup of PCa is usually subjected to active surveillance. Accordingly, in order to avoid unnecessary biopsies, the possibility to assess tumor behavior by using a non-invasive serum test was explored. For this purpose, a data analysis pipeline focused only on PCa group was exploited. In particular, PCa patients were split in two subgroups: 53 AG-PCa and 26 NAG-PCa.

**Fig. 6:**
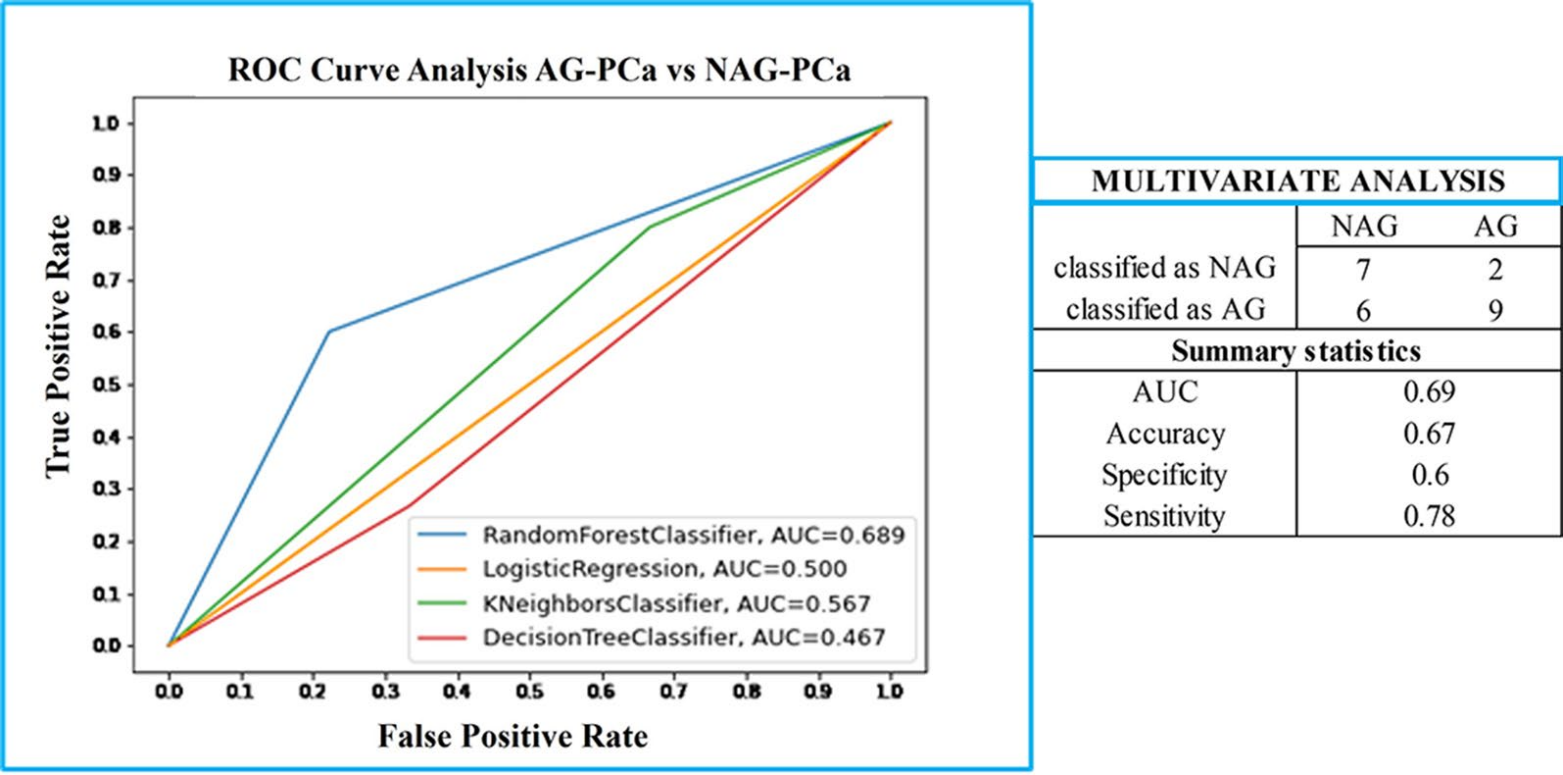
ROC curve analysis of all the 4 tested ML algorithms (Random forest, blue curve; Logistic Regression, orange curve; KNN classifier, green curve; Decision Tree classifier, red curve) and the confusion matrix relative to the best performing ML algorithm (Random Forest)

After feature selection phase, 8 variables were prioritized and used for model testing on 55 samples (70% of the PCa sample set). The 8 variables, ranked on their relative contribution to the model, were: FCN3, proPSA, LGALS3BP, AZU1, C6, LAMB1, CHL1, POSTN. The model was tested with the remaining 30% of the sample set (24 data samples). The best performing model resulted to be Random Forest, which showed a moderate power to discriminate between NAG-PCa and AG-PCa with an AUC of 0.69 (95% confidence interval, CI, 0.57–0.81) (Table 8) (Fig. 6).

**Table 8:** Standard performance metrics for Machine Learning models for the prediction of PCa aggressiveness

Algorithm	AUC	f1	Accuracy	Specificity	Sensitivity
Random forest	0.69 (0.57–0.81)	0.69 (0.57–0.81)	0.67 (0.54–0.79)	0.60 (0.47–0.73)	0.78 (0.67–0.89)
Logistic regression	0.50 (0.37–0.63)	0.77(0.66–0.88)	0.63 (0.50–0.75)	1	0
KNN	0.57 (0.44–0.70)	0.73 (0.61–0.84)	0.63 (0.5–0.75)	0.80 (0.69–0.91)	0.33 (0.21–0.46)
Decision tree	0.43 (0.30–0.56)	0.29 (0.17–0.41)	0.38 (0.25–0.50)	0.20 (0.09–0.31)	0.67 (0.54–0.79)

## Discussion

Blood proteomics is extremely challenging due to the notorious interference of abundant plasma proteins in the detection of lower abundance, tissue leakage proteins. A strategy to lower the limit of detection of LC–MS/MS analysis in blood proteomics is to enrich for specific classes of proteins on the basis of characteristics such as protein size or post-translational modifications. The enrichment of glycopeptides or sialylated glycopeptides has been proposed for over a decade as a means of reaching the detection of proteins present in serum or plasma at concentration levels in the ng/mL range. In this work, 30 mid- to low- abundance proteins carrying at least one glycosylation site, selected on the basis of their potential relevance in PCa progression, have been quantified by relying, for the great majority of analytes, on isotopic dilution for minimizing LC–MS/MS bias. Machine learning algorithms have been applied to a matrix composed of both proteomic and clinical variables, generating a predictive model based on six proteomic variables (RNASE1, LAMP2, LUM, MASP1, NCAM1, GPLD1) and five clinical variables (prostate dimension, proPSA, free-PSA, total-PSA, free/total-PSA). Such model was able to distinguish PCa from BPH patients in an independent set of samples with an AUC of 0.93 (Random Forest model). This value is comparable to previous studies performed on biofluids such as seminal plasma [31], neat urine [32], urine enriched in prostatic secretions [33], and blood plasma [14, 34]. In particular, Drabovic et al. proposed a 2-protein diagnostic panel composed of TGM4 and PAEP gene products to be detected in seminal plasma which could discriminate between negative biopsy and PCa with an AUC of 0.76 (CI 95% 0.74–0.79) [31]. In another work performed on urine enriched in prostatic secretions [33], Kim revealed that a diagnostic panel composed by 6 peptides belonging to five gene products (IDHC, SERA, IGJ, EF2 and KCRB) was able to discriminate between BPH and PCa patients with an AUC of 0.77 (95% CI 0.68–0.87) in a large cohort of samples (n = 207). Jedinak reported a multimodal biomarker combining three proteins (β2M, PGA3 and MUC3) discovered by proteomic analysis of neat urine with PSA to discriminate BPH and PCa in 173 patients, obtaining an AUC of 0.81 (95% CI 0.74–0.89) [32]. Concerning blood serum/plasma analysis, Cima et al., by utilizing glycopeptide capture and targeted LC–MS/MS analysis of de-glycosylated peptides, thus by using a strategy similar to the one employed in this work, have discovered a four-protein signature comprising ASPN, CTSD, HYOU1, OLFM4 which, in combination with PSA provided an AUC of 0.84 (95% CI = 0.82–0.96) in 82 patients [14]. In a follow-up work of this study, undertaken by some of the same authors, THBS1 and CTSD were assayed by ELISA in 474 men suffering from either PCa or BPH [34]. Using a multivariable logistic regression model which also included ftPSA ratio, the authors could discriminate among biopsy-positive and biopsy-negative patients with an AUC of 0.86 (95% CI 0.82–0.91).

The term of comparison for the evaluation of our model performance was the univariate analysis based only on tPSA (measurement routinely performed in the clinic). As it can be seen in Fig. 5, the model based on clinical + proteomic variables (blue line) in our sample cohort for Random Forest algorithm displayed the highest AUC values (0.93). All ML algorithms (Additional file 5), showed AUC values consistently higher than the ones obtained by univariate analysis based on tPSA (red line). Our model was furtherly validated by implementing a voting strategy on 20 patients belonging to the diagnostic grey zone (tPSA between 4 and 10 ng/mL).

This approach, based on the integration of the results of all the of ML algorithms, resulted in the correct assignment of 17/20 samples (85% of the tested samples).

We also explored the possibility of assessing tumor aggressiveness by using proteomic and clinical variables. In this case, feature selection led to six proteomic variables and one clinical variable (proPSA). The classifier based on Random Forest showed a moderate power to discriminate between NAG-PCa and AG-PCa with an AUC of 0.69. This result is comparable to the one obtained by Wang et al. [35] using DIA-MS analysis of nonglycosylated serum proteins for discovery and ELISA assay for validation on two proteins (SPP1 and CP). To address this issue, to date, more promising results have been obtained by performing glycoproteomic analysis on urine as biological specimen, collected either before or after digital rectal exam [36–39]. In these studies, AUC values up to 0.85 have been reported in the effort to classify AG and NAG disease.

## Conclusions

In this work, the development of a multivariate model for discriminating PCa from BPH patients has been described. An initial discovery effort was performed on sample pools and involved the use of isobaric labelling. Two different label-based strategies were adopted, differing only in the timing of the TMT-labeling step. Labeling before glycopeptide enrichment, though costly, dramatically reduced random error. Peptide candidates were assayed by PRM assays on individual samples belonging to a larger cohort. The multivariate model here reported, based on the Random Forest approach, achieving an AUC of 0.93, outperformed the univariate approach relying on tPSA alone. In fact, in our sample set, univariate analysis using tPSA values provided an AUC of 0.79. This latter value, higher than commonly observed, was probably due to the higher serum PSA average levels in the PCa cohort respect to the BPH cohort.

This study, though, has some limitations. In particular, the size of the sample-set prevents drawing a definitive conclusion about the performance of the model and its straightforward applicability in routine. Furthermore, the model comprises formerly N-glycosylated peptides from six different low-abundance proteins. The routinary dosage of these six proteins, by alternative methods such as ELISA, could be laborious to implement and represents another weak point of this model. On the other hand, the PRM assay here developed could provide simultaneous quantification of these six proteins by relying on their corresponding surrogate peptides in a single assay. Five clinical variables were also integrated into the model; some of them, such as tPSA, ftPSA and proPSA are already being used in the clinical routine.

A pilot analysis aimed at separating AG-PCa to NAG-PCa was carried out. However, the developed model of PCa aggressiveness achieved only moderate results showing an AUC of 0.69 with the best performing algorithm (Random Forest). The latter was only an exploratory assessment of the performance of our pipeline to address clinical needs. There is no doubt that the discrimination of two tumoral conditions, albeit characterized by different aggressiveness, presupposes small differences that require larger datasets. Therefore, more extensive studies are needed in larger cohorts of patients.

### Supplementary Information

**Additional file 1**: **Discovery experiments: Discovery TMT-A** (Glycopeptide enrichment; TMT labelling, Strong cation exchange (SCX) StageTip) **Discovery** **TMT-B** (Sample desalting by solid-phase extraction (SPE); High pH C_18_ fractionation), **LC-PRM assay development **(LC-PRM acquisition method). **Verification experiments:** Sample processing workflow; Ultimate LC-PRM acquisition method.

**Additional file 2**: **Table S1.** List of heavy peptides assayed by PRM with relative retention times (RT) and solubilization conditions. **Table S2.** List of heavy peptides used in the verification experiments and relative concentration in the heavy peptide mixture (HPM). **Table S3.** LC-MS/MS PRM method for the acquisition of 163 samples. **Table S4.** Peptides used as normalization factors

**Additional file 3**: **Table S5.** Sum of all XIC values for the 30 reference glycopeptides used as normalization factors in all replicates.

**Additional file 4:** Peptide intensities (**Table S6**) and clinical parameters (**Table S7**) acquired for the samples in the data set.

**Additional file 5**: Peptide identification and quantification in discovery experiments TMT-A and TMT-B. PCa sample pools have been labelled with isobaric labels 126-128, whereas BPH sample pools were labelled with isobaric labels 129-131.

**Additional file 6**: Confusion matrixes, confidence intervals and ROC curves for the remaining four ML algorithms (KNN, SVM, Logistic regression, Decision Tree). The following sets of variables were considered: (i) proteomic + clinical (named “Multivariate Analysis”, blue), (ii) proteomic variables only (named “Peptides Analysis”, green), (iii) clinical variables only (named “Biological Samples”, red), (iv) PSA only (named “Univariate Analysis”, red).

## Data Availability

The mass spectrometry proteomics data have been deposited to the ProteomeXchange Consortium (http://proteomecentral.proteomexchange.org) via the PRIDE partner repository with the dataset identifier PXD035935. Reviewer account details: (i) reviewer_pxd035935@ebi.ac.uk (Username), (ii) 9rCga4X9 (Password).

## Abbreviations

PSA: Prostate-specific antigen
BPH: Benign prostatic hyperplasia
PCa: Prostate cancer
PRM: Parallel reaction monitoring
LC: Liquid chromatography
TiO_2_: Titanium dioxide
ROC: Receiver operating characteristic
MS: Mass spectrometry
NAG: Indolent prostate cancer
AG: Aggressive prostate cancer
SPEG: Solid-phase extraction of N-linked glycopeptides
SWATH: Sequential window acquisition of all theoretical mass spectra
ELISA: Enzyme-linked immunosorbent assay
AUC: Area under the curve
TEAB: Triethylammonium bicarbonate
DOC: Sodium deoxycholate
DTT: Dithiothreitol
IAA: Iodoacetamide
RT: Room temperature
TFA: Trifluoroacetic acid
ACN: Acetonitrile
TMT: Tandem mass tags
FA: Formic acid
nLC-MS/MS: Manoliquid chromatography-tandem mass spectrometry
SPE: Solid-phase extraction
DDA: Data-dependent mode
nESI: Nanoelectrospray
HPM: Heavy peptide mixture
XIC: Extracted ion chromatogram
SRD: Scaled relative difference
tPSA: Total PSA
ftPSA: Free/total PSA
fPSA: Free PSA
KNN: K-nearest neighbours
SVM: Support Vector Machine
LAMP2: Lysosome-associated membrane glycoprotein 2
LUM: Lumican
NCAM1: Neural cell adhesion molecule 1
GPLD1: Phosphatidylinositol-glycan-specific phospholipase D
RNASE1: Ribonuclease pancreatic
MASP1: Mannan-binding lectin serine protease 1
