# Supplementary material for: Development of a predictive model to distinguish prostate cancer from benign prostatic hyperplasia by integrating serum glycoproteomics and clinical variables
Source: Clin Proteomics. 2023 Nov 21;20:52. doi: 10.1186/s12014-023-09439-4 (PMC10662699; doi:10.1186/s12014-023-09439-4)
Supplement: Supplementary file 1 — Additional file 1: Discovery experiments: Discovery TMT-A (Glycopeptide enrichment; TMT labelling, Strong cation exchange (SCX) StageTip) Discovery TMT-B (Sample desalting by solid-phase extraction (SPE); High pH C18 fractionation), LC-PRM assay development (LC-PRM acquisition method). Verification experiments: Sample processing workflow; Ultimate LC-PRM acquisition method. [file 12014_2023_9439_MOESM1_ESM.docx]

**Discovery Experiments**

***Discovery TMT-A***

*Glycopeptide enrichment*

Sample pools (about 160 µg x 4 = 640 µg of serum proteins per pool) were supplemented with trifluoroacetic acid (TFA) to a final concentration of 0.5% (v/v) in order to precipitate DOC. The detergent was pelleted by centrifugation at 13400 rpm for 5 min. The supernatant, containing the peptides, was separated from the DOC pellet, transferred into clean tubes and dried by vacuum centrifuge. Peptides were resuspended in 1 mL of Titanium Loading Buffer (1 M glycolic acid in 80% acetonitrile (ACN)/5% TFA (v/v). Then, after the addition of 2 mg of beads (Titanosphere 5 μm, GL Sciences, Tokyo, Japan), incubation at room temperature (RT) was performed (30 min, 1200 rpm). Beads were subjected to sequential washes to remove non-glycosylated peptides. In particular: one wash with 100 µL of Titanium Loading Buffer, two washes with 100 µL of Titanium Wash 1 (80% ACN/1%TFA), two washes with 100 µL of Titanium Wash 2 (20% ACN/0.1%TFA). Then, the beads were dried and 80 µL of Titanium Eluent solution (1% ammonium hydroxide) were added (15 min, 650 rpm, RT). After incubation, the supernatant (containing glycopeptides) was recovered by 2 min centrifugation at 4000 rpm. Then, glycopeptides were dried and resuspended in 25 µL of 50 mM TEAB and de-glycosylated by overnight incubation with 1 µL of PNGase F (500 U/µL) (New England Biolabs, Ipswich,MA) (650 rpm, 37 °C).

*TMT labelling*

TMT labelling was performed following the manufacturer’s protocol except for the resuspension volume of TMT reagents, which was 100 µL of anhydrous ACN (final TMT concentration of 0.8 µg/µL). Each pool was labelled with a different TMT channel using only 10 µL of the stock solution. Samples were incubated at RT for 60 min at 800 rpm. Then, the labelling reaction was quenched by using 0.8 µL of 5% Hydroxylamine (w/v). TMT-labelled sample pools were combined in 1:1 ratio into a single sample. This sample was 5-fold diluted in Wash B (80% ACN/0.5% formic acid (FA) (v/v)) and then fractionated by strong cation exchange (SCX) StageTip

*Strong cation exchange (SCX) StageTip*

SCX fractionation was performed by inserting 1 plug of Empore^TM^-3M SCX resin, withdrawn using a blunt-ended syringe needle (gauge 16), into a 200 µL pipette tip. Briefly, the StageTip was conditioned with 50 µL of SCX Wash A (20% ACN/0.5% FA(v/v)) and equilibrated with SCX Wash B. Then, the sample mix was carefully loaded into the resin. Sample loading was followed by consecutive washes with SCX Wash B and SCX Wash A. Peptides were stepwise eluted by adding 10 µL of six separate eluent solutions of increasing ionic strength. Particularly, eluents were: 20% ACN/0.5% acetic acid (v/v) and (I) 50 mM, (II) 75 mM, (III) 100 mM, (IV) 150 mM, and (V) 250 mM ammonium acetate, respectively. The last eluent (VI) was composed of 20% ACN (v/v) and 500 mM ammonium acetate. Then, the 10% of each fraction was analysed by nanoliquid chromatography-tandem mass spectrometry (nLC-MS/MS).

***Discovery TMT-B***

*Sample desalting by solid-phase extraction (SPE)*

Twenty-five µL of digested samples (about 40 µg) were pooled in groups of 4 samples for a total of 10 pooled samples (5 PCa and 5 BPH). Subsequently, each sample was diluted with 100 mM TEAB to 800 µL. Then, 320 µL of TMT-10 plex solution (prepared by diluting 5 mg of TMT reagent with 625 µL of anhydrous ACN) were added. After labelling, each channel contained about 160 µg of peptides. After having verified that the labelling reaction was complete, by injecting a small aliquot of each sample in nLC-MS/MS prior to quenching,(19) the labelling reaction was quenched by hydroxylamine. Then, all samples were combined in 1:1 ratio into a single sample mix (about 1.6 mg in a volume of 12 mL).

The sample mix was acidified by adding 28.8 mL of 0.3% TFA (v/v) to achieve DOC precipitation (centrifugation at 13400 rpm 5 min). Then, the supernatant was collected. Before TiO_2_ enrichment, the sample was split into four aliquots and desalted on four separate solid-phase extraction (SPE) HLB 1 cc cartridges (Waters, 10 mg). The cartridges (Waters, HLB 1 cc cartridges 10 mg) were equilibrated by two washes with 1 mL of SPE Solution A (80% ACN/0.1% FA (v/v)) and conditioned by three washes with 1 mL of SPE Solution B (0.1% TFA (v/v)). Then, the peptide mix was 2-fold diluted in H2O and loaded. Two washes with 1 mL of SPE Solution C (0.1% FA (v/v)) were performed before peptide elution by 500 µL of SPE Solution A. The eluates of the 4 cartridges (2 mL total volume) containing about 1.6 mg of peptides were combined and evaporated.

By virtue of a higher quantity of peptide starting material, TiO2 enrichment was performed using 10 mg of beads. Washings and elution were performed as previously described, section “Glycopeptide enrichment”. Then, glycopeptides were de-glycosylated by the addition of 6 µL of PNGase F (overnight incubation at 37 °C with gentle agitation).

Formerly glycosylated peptides were fractionated by C18 (EmporeTM-3M, C18) StageTips performed at basic pH (see below).

*High pH C_18_ fractionation*

Two StageTips (each bearing 2 disks of C_18_ resin, EmporeTM-3M) were conditioned with 50 µL of C_18_ Solution A (50% ACN/0.1% TFA (v/v)) and equilibrated with 50 µL of C_18_ Solution B (0.1% TFA (v/v)). Then, the acidified sample (after the addition of TFA to a final concentration of 0.5% (v/v)) was loaded. After sample loading, a wash with 50 µL of C_18_ Solution B was performed. Peptides were stepwise eluted in ten fractions by the sequential use of eluents with increasing organic content. More specifically, eluents contained 10 mM TEAB/0.12% ammonium hydroxide and increasing amounts of ACN (3%, 5%, 8%, 10%, 12%, 14%, 16%, 20%, 25%, 60%). All fractions were evaporated and resuspended in 16 µL of mobile phase A (2% ACN/0.1% FA (v/v)). Then, 25% of each fraction was analysed by nLC-MS/MS.

**LC-PRM assay development**

*LC-PRM acquisition method*

Chromatographic separation was performed by nanoflow chromatography using EASY-LC-1000 instrument (Thermo Fisher) coupled with a Q-Exactive mass spectrometer (Thermo Fisher). Peptides were separated by an in-house made analytical column packed to 14 cm of length with 3μm C18 silica particles (Dr. Maisch). Gradient elution was obtained using a binary gradient at a flow rate of 300 nL/min. The mobile phase A and B were (2% ACN/0.1% FA (v/v)) and (80% ACN/0.1% FA (v/v)) respectively. The percentage of mobile phase B started at 12%, then moved to 40% in 60 min and to 100% in 6 min. After 10 min at 100%, mobile phase B was then decreased to 0% in 4 minutes. Ionization of peptides gradually eluting from the analytical column was performed by nESI, applying a potential of 1700 V to the column injection-end via a tee piece.

PRM acquisition was performed as follows: a full MS event (scan range 350-1800 *m/z*) at a resolution of 17500, was followed by targeted MS/MS events performed at a resolution of 35000. AGC target and maximum injection time for full MS and PRM were 5×10^5^/2×10^5^ ions, 200/120 ms, respectively. Mass window for precursor ion isolation was 1.6 *m/z*. Normalized collision energy was 25. Ion threshold for triggering MS/MS events was 1×10^5^. Dynamic exclusion was 30 s.

**Verification experiments**

*Sample processing workflow*

Protein digestion, the quantity of digested proteins was reduced to 640 µg. Buffer volumes and trypsin amount were diminished accordingly (-20%). DOC was precipitated by adding 5% TFA (final concentration) and by separating the supernatant (95% of volume) after having performed a centrifugation step lasting 5 min at 13400 rpm. For what concerns glycopeptide enrichment and de-glycosylation, peptide mixtures were completely dried, and N-glycopeptides were enriched as illustrated in the section Discovery Experiments by the use of 2 mg of titanium beads for each sample. Glycopeptides were eluted from beads by 80 µL of 1% ammonium hydroxide, lyophilized and resuspended in 50 µL of 100 mM TEAB. Then, de-glycosylation was achieved by incubating samples with 1 µL of PNGase F (overnight incubation at 37 °C). The, HPM (stored at -80 °C in aliquots of 10 µL) was thawed, diluted with 165 µL of 0.25% TFA and added to the peptide mixtures obtained after PNGase incubation. The resulting solution was subjected to C_18_ purification as previously described. Before nLC-MS/MS analysis, samples were completely dried and resuspended in 15 µL of solution A (2% ACN/ 0.1% FA); 4 µL were analysed by LC-MS/MS in PRM mode. The final protocol required the digestion of only 8 µL of serum as starting material. After glycopeptide enrichment, we had a peptide yield of about 1.6 µg. Then, ¼ of the enriched sample containing about 400 ng of peptides, was analysed by PRM.

This whole pipeline was carried out in duplicate for 84 BPH and 79 PCa serum samples, accomplishing a total of 326 nLC-MS/MS analyses.

*Ultimate LC-PRM acquisition method*

Gradient was from 0% to 3% B in 1 s, from 3% to 36% B in 35 min and then to 40% B in 10 min, finally to 100% in 5 min. After 5 min at 100%, mobile phase B was then decreased to 0% in 5 minutes. MS detection of peptides gradually eluted from the analytical column, was performed by nESI applying a potential of 1700 V to the column front-end via a tee piece.

Multiplexed target acquisition was performed as follows: a full MS event (scan range 350-1800 *m/z*) at a resolution of 17500 was followed by targeted MS/MS scans divided in acquisition windows as follows: (1) from 0 to 20.7 min, 5 MS/MS events performed at a resolution of 35000; (2) from 20.7 to 33.01, 10 MS/MS events performed at a resolution of 17500; (3) from 33 to 60, 5 MS/MS events performed at a resolution of 35000. AGC target for full MS was 5×10^5^, for all PRM scans was 2×10^5^. Maximum injection time for full MS was 200 ms, for PRM (1) and (3) was 120 ms, for PRM (2) was 65 ms. Mass window for precursor ion isolation was 1.6 *m/z*. Normalized collision energy was 25. A schematic view of the PRM method is reported in Additional file 2 (Table S3).
